# Developing an operational definition of housing instability and homelessness in Veterans Health Administration’s medical records

**DOI:** 10.1371/journal.pone.0279973

**Published:** 2022-12-30

**Authors:** Jack Tsai, Dorota Szymkowiak, Eric Jutkowitz

**Affiliations:** 1 VA National Center on Homelessness among Veterans, Tampa, FL, United States of America; 2 School of Public Health, University of Texas Health Science Center at Houston, Houston, TX, United States of America; 3 Department of Psychiatry, Yale University School of Medicine, New Haven, CT, United States of America; 4 Center of Innovation in Long Term Services and Supports, Providence VA Medical Center, Providence, RI, United States of America; 5 Department of Health Services, Policy & Practice, Brown University School of Public Health, Providence, RI, United States of America; Centre for Addiction and Mental Health, CANADA

## Abstract

The main objective of this study was to examine how homelessness and housing instability is captured across data sources in the Veterans Health Administration (VHA). Data from 2021 were extracted from three data repositories, including the Corporate Data Warehouse (CDW), the Homeless Operations Management System (HOMES), and the Homeless Management Information System (HMIS). Using these three data sources, we identified the number of homeless and unstably housed veterans across a variety of indicators. The results showed that the use of diagnostic codes and clinic stop codes identified a large number of homeless and unstably housed veterans, but the use of HOMES and HMIS data identified additional homeless and unstably housed veterans to provide a complete count. A total of 290,431 unique veterans were identified as experiencing homelessness or housing instability in 2021 and there was regional variability in how homelessness and housing stability were captured across data sources, supporting the need for more uniform ways to operationalize these conditions. Together, these findings highlight the and encourage use of all available indicators and data sources to identify homelessness and housing instability in VHA. These methodologies applied to the largest healthcare system in the U.S. demonstrate their utility and possibilities for other healthcare systems. Transparent practices about data sources and indicators used to capture homelessness and housing instability should be shared to increase uniform use.

## Introduction

Homelessness is a major public health problem in the U.S. that affects hundreds of thousands of Americans annually [1–3]. People who experience homelessness are at increased risk of death and disproportionally rely on acute care [4–6]. Because homelessness affects health and healthcare utilization, healthcare systems have developed interventions to prevent homelessness and provide care for people experiencing homelessness [7, 8]. Effective interventions and research on these interventions require the ability to accurately identify people experiencing homelessness.

However, one challenge with addressing homelessness in healthcare systems is difficulties with coding and operationalizing homelessness in medical records [9]. Conceptually, homelessness is not a medical condition and there is no specific diagnostic code in the Diagnostic and Statistical Manual of Mental Disorders, Fifth Edition (DSM-5) or the International Classification of Diseases, Version 10 (ICD-10) beyond descriptive codes such as DSM-5 V-codes and ICD-10 Z codes. Thus, many healthcare systems rely on some combination of screeners, descriptive diagnostic codes that are not uniformly used, or documentation of homeless status in free-form narrative notes. Furthermore, providers may not consistently or uniformly document homelessness in the medical record.

There are several important reasons to examine ways to identify and document homelessness and housing instability in the Veterans Health Administration (VHA). For one, it is national tragedy that many veterans who served the country experience homelessness and many risk factors for veteran homelessness have been discovered [10]. Secondly, preventing and ending homelessness among veterans has been a stated priority of VHA for over a decade and considerable progress has been made on that front including improved outreach, program development, and new research- and these efforts have helped advance new ways to capture homelessness [11, 12]. Thirdly, VHA operates the largest integrated healthcare system in the U.S. with a national healthcare record that provides a unique opportunity to study a large number of medical records across the country and examine experiences of both housing instability and homelessness. Fourthly, accurately identifying veterans experiencing homelessness allows the VHA and other healthcare systems to evaluate outcomes of their investments in homeless programs.

Previous studies have used city, state, healthcare system, or VHA data to determine strategies to identify people experiencing homelessness. One popular approach used by researchers is to rely on the address provided by a patient and checking if the address registered as a hospital, homeless shelter, or place of worship [13, 14]. Using addresses to identifying homelessness has limitations. Relying on historical address data to identify housing status does not account for transitions in housing. Importantly, there are technical and practical challenges with using address data including variation in spelling of addresses, limits in data sharing across healthcare providers/systems, and exclusion of homeless individuals who provide no addresses.

Within the VHA, researchers commonly identify veterans experiencing homelessness via ICD-9/10 V/Z codes, specifically ICD-9 codes V60.0 (“lack of housing”) and V60.1(“inadequate housing”) and ICD-10 codes Z59.0 (“homelessness”) and Z59.1 (“inadequate housing”) [15–17]. Other V/Z codes may also be used by providers as a proxy for homelessness. For example, V60.2 is a code to indicate “inadequate material resources.” In addition to diagnostic codes, VHA studies have also defined homelessness by receipt of any homeless program services as documented in administrative records [15, 16]. A prior study that examined how homelessness is coded in the VHA found variation across medical centers and no uniform method [17]. Importantly, the study did not include data from VHA’s Homeless Operations Management Evaluation System (HOMES) which has become the main homeless database that captures data from VHA homeless programs. We also thought it important to conceptualize housing instability and homelessness as two separate, but related constructs. We defined *housing instability* as the state of living in housing but currently being at-risk of losing that housing; in contrast, we defined *homelessness* as the state of living in a place not meant for human habitation (e.g., outdoors, vehicles), in emergency shelter, in transitional housing, or exiting an institution (hospital, jail) with no permanent housing arrangement.

To provide contemporary examination on this topic with the purpose of developing “best practices” in operationalizing homelessness, we conducted a methodological study using different data sources and indicators available through the VHA system. Specifically, we sought to identify the numbers of veterans experiencing homelessness and housing instability from different VHA data sources across the country and with respect to geographic location. We hypothesized using all available VHA data sources would yield much greater identification of homeless and unstably housed veterans than limiting data sources, and that there would be wide variability in use of different indicators of homelessness and housing instability by region. These findings may inform providers in the VHA system, guide researchers on ways to identify homeless and unstably housed veterans, and shape administration and policies in local and regional healthcare systems inside and outside VHA to improve identification of homelessness.

## Methods

Data used to identify veterans experiencing housing instability and homelessness reside in three national repositories accessible to authorized VHA users. These three data repositories are: Corporate Data Warehouse (CDW), which contains the entire VHA medical record populated by providers during clinical encounters; Homeless Operations Management System (HOMES), which contains data about veterans’ participation in nearly all VHA homeless programs populated by homeless service staff; and Homeless Management Information System (HMIS), which is not a VHA database and is populated by community grantees but contains national data about veterans’ participation in VHA’s Supportive Services for Veteran Families (SSVF) program. This was a quality improvement project, need for participant consent was waived, and this work was considered exempt from the institutional review board.

Tables 1 and 2 outline the different data sources, codes, and descriptions that were used to operationalize housing instability and homelessness in VHA. Using data from calendar year 2021, we conducted frequency analyses in a cascading manner (i.e., the additional identification of veterans with each additional indicator) in order to describe the number of veterans identified as experiencing housing instability or homelessness with each subsequent data source and code. We also examined overlap between the numbers of veterans identified as experiencing housing instability and veterans identified as experiencing homelessness in 2021 by capturing the number of unique veterans who experienced *either* housing instability or homelessness, and *both* housing instability and homelessness in the same year. Lastly, to understand geographic variability in use of these codes, we conducted analyses to examine use of different codes for housing instability and homelessness by VHA’s designated catchment areas called Veterans Integrated Service Networks (VISNs). There are 18 VISNs covering different regions throughout the U.S. Analyses by VISN allows comparisons between geographic regions and examination of how consistently different codes are used.

**Table 1:** Data source, codes, and descriptions for defining housing instability in Veterans Health Administration medical records.

Data source	Codes	Description
Corporate Data Warehouse (CDW)	International Classification of Diseases (ICD) diagnostic codes Z59.1, Z59.81 in inpatient and outpatient records	Descriptive diagnostic codes indicating “inadequate housing” or “housing instability.”
CDW	A positive screen on part 1 of two-part annual homeless screening clinical reminder (HSCR)	A “yes” response to the question: “Are you worried or concerned that in the next two months you may NOT have stable housing that you own, rent, or stay in as part of a household?”
Homeless Management Information System (HMIS)	Record of participation in the Supportive Services for Veteran Families (SSVF) program	The SSVF program includes services for rapid rehousing and homeless prevention and is provided by VHA-funded grantees.
CDW	Outpatient stop codes 507, 522, 530	Clinical encounters with staff in the Housing and Urban Development-Veterans Affairs Supportive Housing (HUD-VASH) program.
Homeless Operations, Management and Evaluation System (HOMES)	Record of participation in the HUD-VASH program	The HUD-VASH program involves a rental subsidy provided by HUD and case management services provided by VHA.

Note: The greyed areas indicate data sources and codes that are generally not recommended as indicators of housing instability unless a justification is provided to include HUD-VASH.

**Table 2:** Data sources and definitions for homelessness from the VA medical record.

Data source	Codes	Description
Corporate Data Warehouse (CDW)	International Classification of Diseases (ICD) diagnostic code Z59.0 in inpatient and outpatient records	Descriptive diagnostic codes indicating “homelessness.”
CDW	Outpatient stop codes 504, 507, 508, 511, 522, 528, 529, 530, 555, and 556. Inpatient specialty codes 28, 29, 37, and 39.	Clinical encounters with outpatient staff in the Grant and Per Diem (GPD) program and the Health Care for Homeless Veterans (HCHV)/Homeless Chronically Mentally Ill (HCMI) program. Clinical encounters with inpatient staff included the Domiciliary Care for Homeless Veterans (DCHV) program and the Compensated Work Therapy/Transitional Residence (CWT/TR) program.
Homeless Operations, Management and Evaluation System (HOMES)	Record of participation in GPD, HCHV/HCMI, DCHV, or CWT/TR.	The GPD provides transitional housing in the community and the DCHV provides transitional housing at VHA facilities. The HCHV/HCMI program provides short-term case management and temporary housing. The CWT/TR program provides vocational rehabilitation and transitional housing.
CDW	A positive screen on part 2 of a two-part annual Homeless screening clinical reminder (HSCR).	A “no” response to the question: “In the past two months, have you been living in stable housing that you own, rent, or stay in as part of a household?”

## Results

**Fig 1:**
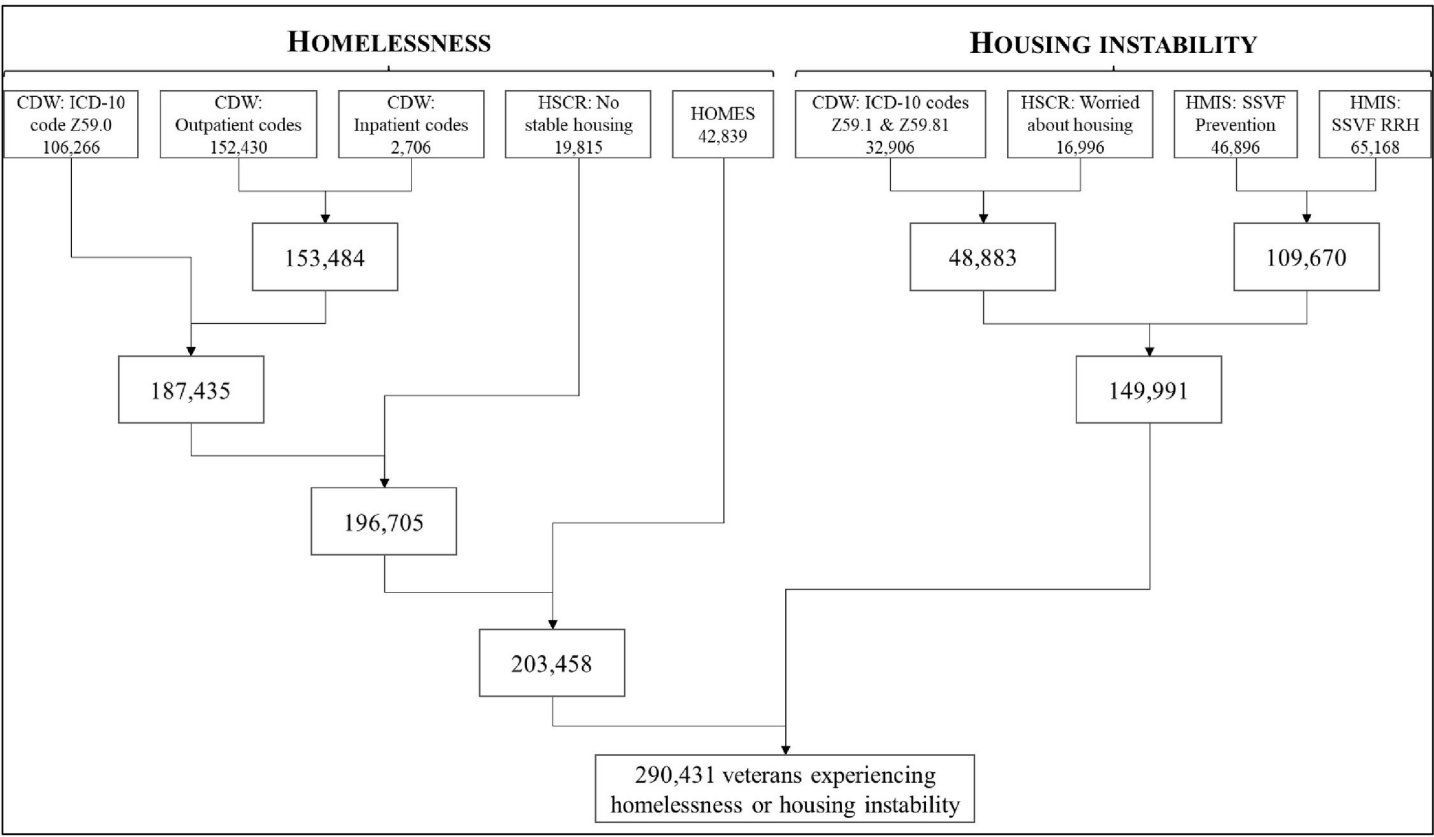
Numbers of homeless and unstably housed veterans identified with different data sources. Note: CDW = Corporate Data Warehouse; ICD-10 = International Classification of Diseases, Version 10; HSCR = Homeless Screening Clinical Reminder; HOMES = Homeless; HMIS = Homeless Management Information System; SSVF = Supportive Services for Veterans Families, RRH = Rapid Re-housing.

Using 2021 data, we describe the number of unique veterans identified as experiencing housing instability and the number of unique veterans identified as experiencing homelessness using different data sources and codes (Fig 1). In identifying veterans who experienced housing instability, the use of ICD-10 codes and outpatient stop codes extracted from CDW identified a large number of veterans. But each additional indicator and data source that was used, including the HSCR extracted from CDW, HOMES records, and SSVF data from HMIS, helped identify additional veterans experiencing housing instability that would not have been identified otherwise. We did not consider participation in the HUD-VASH program as an indicator of housing instability, but the S1 Fig presents the results if the HUD-VASH program is included.

In identifying homeless veterans, we found that the largest number of veterans could be identified by outpatient and inpatient stop codes but ICD-10 codes, the HSCR, and HOMES records had incremental value in identifying homeless veterans. For example, 153,484 unique homeless veterans were first identified with CDW outpatient and inpatient clinic stop codes, and then 33,951 additional unique veterans were identified with CDW ICD-10 codes. After that, 9,270 more unique homeless veterans were identified with the inclusion of the HSCR and a final 6,753 unique homeless veterans were identified resulting in a total of 203,458 unique veterans identified across the homeless indicators.

There was overlap between the number of veterans we identified as experiencing homelessness and the number of veterans who identified as experiencing housing stability. Our calculations revealed a total of 290,431 unique veterans were identified as experiencing housing instability *or* homelessness in 2021; and a total of 63,018 unique veterans were identified as having experienced homelessness *and* housing instability in 2021.

As shown in Table 3, there was notable variability between VISNs in the use of different codes to identify homelessness among veterans. For example, identifying homeless veterans using ICD-10 codes captured 41% to 62% of all homeless veterans identified within each VISN. However, there were also some consistent patterns observed. The use of outpatient clinic codes identified the highest proportion of homeless veterans within each VISN, while use of inpatient clinic stop codes identified the lowest proportion of homeless veterans within each VISN. HOMES data (which excluded HUD-VASH records) captured 34–53% of homeless veterans identified within each VISN and supplemented other data sources such as outpatient codes in providing a complete count of homeless veterans. No one data source or code captured all the homeless veterans identified in each VISN, but with the data sources combined a total of 203,458 homeless veterans were identified varying from 6,036 in VISN 15 to 24,240 in VISN 22. Across VISNs, homeless veterans constituted about 2.4% of all veterans (ranging from 1.4%-3.6% within each VISN).

**Table 3:** Geographic variability in use of different indicators of homelessness.

Veterans Integrated Services Network (VISN)	ICD-10 diagnostic codes % of homeless veterans in VISN	Outpatient clinic stop codes % of homeless veterans in VISN	Inpatient clinic stop codes % of homeless veterans in VISN	HSCR % of homeless veterans in VISN	Participation in homeless programs recorded in HOMES % of homeless veterans in VISN	Total number of unique homeless veterans identified across indicators	% of total unique homeless veterans in VISN
1	48.8%	75.3%	2.5%	10.1%	25.3%	7,697	2.4%
2	44.8%	72.4%	0.5%	8.5%	22.1%	9,027	2.2%
4	47.5%	78.6%	1.8%	7.8%	23.9%	9,289	2.5%
5	48.5%	75.7%	0.5%	10.6%	19.5%	6,976	2.3%
6	50.7%	81.1%	0.3%	9.3%	12.5%	11,447	2.2%
7	43.9%	77.3%	2.4%	8.7%	13.1%	16,632	2.6%
8	62.1%	66.1%	1.2%	9.1%	20.9%	17,600	2.7%
9	57.0%	75.0%	0.0%	8.4%	21.6%	7,458	2.3%
10	46.4%	78.6%	2.7%	7.5%	27.5%	13,585	2.3%
12	40.6%	82.5%	0.3%	6.1%	24.8%	8,695	2.2%
15	50.9%	75.2%	2.0%	7.7%	23.3%	6,036	1.4%
16	52.1%	74.2%	0.5%	8.2%	13.0%	14,236	2.8%
17	48.2%	71.7%	0.5%	12.5%	19.6%	12,422	2.3%
19	56.9%	70.3%	1.2%	10.4%	15.3%	10,825	2.6%
20	57.8%	66.8%	1.3%	8.6%	23.5%	11,516	2.7%
21	58.8%	76.2%	0.3%	9.0%	25.4%	16,276	3.6%
22	57.2%	73.4%	2.3%	13.7%	22.2%	25,240	3.5%
23	43.2%	78.4%	0.8%	6.9%	24.3%	6,264	1.7%
Total (across VISNs)	52.2%	74.9%	1.3%	9.7%	21.1%	203,458	2.4%

Note: A map of VISN locations can be viewed here: https://www.va.gov/HEALTH/visns.asp ICD-10 = International Classification of Diseases, Version 10; HSCR = Homeless Screening Clinical Reminder; HOMES = Homeless Operations Management Evaluation System.

There was also geographic variability between VISNs in the use of codes to identify housing instability among veterans as shown in the S1 Table. Housing instability was predominantly identified with outpatient clinic stop codes, HOMES data, and HMIS data; and only small proportions of veterans were identified as unstably housed with ICD-10 codes and the HSCR in somewhat contrast to the findings on codes for homelessness. Together, with the use of all indicators across data sources, a total of 149,991 unstably housed veterans were identified varying from 4,598 to 16,914 across VISNs. In both identifying housing instability and homelessness, the analyses by VISN found some geographic areas used some indicators more than others but use of all indicators mitigates the geographic variability.

## Discussion

In this methodological study, we demonstrate the various data sources and indicators that can be used to identify veterans experiencing housing instability and/or homelessness. The findings may guide researchers studying homelessness in VHA, inform stakeholders that rely on VHA research on homelessness veterans, and provide models to record and identify homelessness in other systems outside of VHA. While we provide a comprehensive multi-source method to identify homeless and unstably housed veterans, some researchers may choose to utilize only a few of these data sources and indicators. However, it may be considered best practice to utilize all the indicators described in this study across the three data sources (CDW, HOMES, HMIS) to fully capture housing instability/homelessness and capitalize on the VA’s unique administrative medical record system. Our examination described how many homeless veterans were identified in each data source and indicator so proper caveats can be considered when only a few data sources/indicators are used. For example, data from this study can provide estimates of how many homeless veterans may have been missed in the analysis if a data source/indicator was not used or justify its exclusion.

This study highlights that VHA has various ways to capture data on housing instability and homelessness which may reflect its decades-long focus on preventing and ending veteran homelessness. The VHA system that exists now represents an accumulation of resources and knowledge over time that may benefit the broader homeless service field. VHA’s main database for homeless programs is now HOMES and the data show that many homeless and unstably housed veterans can be identified without HOMES but the inclusion of other indicators with HOMES data consistently provides the most complete count of veterans. This study extends the work of a previous study that did not use HOMES data at all [17]. HOMES is an important resource that provides incremental value in identification of homelessness and housing instability among users in the VHA system. In addition, our analysis was focused on identifying veterans experiencing homelessness or housing instability with ICD-10 diagnostic codes and clinic stop codes capturing more cases, but these data sources lack important contextual information (e.g., type of housing problem, use of homeless programs) that is available in HOMES.

There was geographic variability in the ways that different VHA catchment areas documented housing homelessness and housing instability, and there is no system-wide effort to train providers on how to capture homelessness. However, while there was variability, many VHA catchment regions captured homelessness in similar ways, with outpatient stop codes being the most common followed by ICD-10 codes and inpatient stop codes being the least common. Notably, less than 15% of homeless veterans were identified by the HSCR which is a system-wide VHA screening that has been implemented to screen for homelessness. By implication, these findings suggest more systematic use of the HSCR along with ICD-10 codes which are not tied to clinical workload (like stop codes are) may be warranted.

It may be important to note that the number of veterans we identified as experiencing homelessness was higher than the number identified as experiencing housing instability. This is likely an artifact of our operationalization of these conditions and the limits of the data sources and indicators available. In reality, it is a near certainty that the population of unstably housed veterans is larger than the population of homeless veterans. This discrepancy reflects challenges that healthcare systems face, including the VA, in identifying unstably housed patients since homelessness is a more distinct category with urgent clinical implications whereas housing instability is a broader more nebulous construct that may not be as easily and commonly assessed in clinical practice and subsequently coded in medical records. There may be ways to improve identification of housing instability, such as more widespread use of screeners like the HSCR, better training for providers on how to screen and identify housing instability, and more open ways for patients to self-identify or self-refer for housing assistance. There are also new advanced technologies such as natural language processing that can mine free-text provider notes to detect cases of housing instability [18, 19] and may present new ways to extract information that may be relevant to researchers and providers.

Our analyses had several limitations worth noting. We did not have a gold standard reference or independently verified cases of homelessness to compare our findings against. This was a methodological study, so we did not focus on prevalence rates, associations between variables, or level of VHA eligibility and VHA benefits which may be important to understand; but our study provides foundation for these future areas for study. Analyses were restricted to veterans who were enrolled and receiving VHA services. Finally, our findings are specific to VHA, which has a particularly comprehensive medical record system and operates a continuum of homeless programs across the U.S. However, our findings may inform ways to code and operationalize homelessness and housing instability in other healthcare systems.

In conclusion, ICD-10 and outpatient/inpatient clinic stop codes capture the majority of documented cases of homelessness and housing instability, but do not provide a full count. These codes are easily accessible to researchers by querying the VHA electronic medical record. Using the housing screener, HOMES and HMIS marginally increases the number of cases and provide more rich data for context. As providers, researchers, policymakers, and other stakeholders interpret findings regarding homeless and unstably housed veterans using VA data, it is important to recognize the structural characteristics of how homelessness and housing instability is documented in various VHA databases. The findings may also inform ongoing efforts to transform the VHA medical record system to the Cerner Millennium platform and stimulate development of innovative data capture methods to operationalize homelessness and housing instability in broader healthcare fields interested in addressing social determinants of health.

## Supporting information

S1 Fig Numbers of unstably housed veterans identified with different data sources, treating participation in HUD-VASH as an indicator of housing instability. Note: ICD = International Classification of Diseases; HSCR = Homeless Screening Clinical Reminder; HUD-VASH = Housing and Urban Development-Veterans Affairs Supportive Housing, HOMES = Homeless Operations Management Evaluation System, SSVF = Supportive Services for Veterans Families, RRH = Rapid Re-housing.
(DOCX)

S1 Table Geographic variability in use of different indicators of housing instability. Note: A map of VISN locations can be viewed here: https://www.va.gov/HEALTH/visns.asp ICD-10 = International Classification of Diseases, Version 10; HSCR = Homeless Screening Clinical Reminder; HOMES = Homeless Operations Management Evaluation System; SSVF = Supportive Services for Veteran Families; HMIS = Homeless Management Information System.
(DOCX)
