# Correction: Developing an operational definition of housing instability and homelessness in Veterans Health Administration’s medical records

**DOI:** 10.1371/journal.pone.0322834

**Published:** 2025-04-24

**Authors:** Jack Tsai, Dorota Szymkowiak, Eric Jutkowitz

## Introduction

In Table 2, Row 2, Column 2, the outpatient stop codes 507, 522, and 530 were mistakenly included. Additionally, in Row 2, Column 3, the authors have added text referencing the Homeless Veterans Community Employment Services (HVCES) program, which is already represented by the stop codes. Please see the correct Table 2 here.

**Table 2:** Data sources and definitions for homelessness from the VA medical record.

Data source	Codes	Description
Corporate Data Warehouse (CDW)	International Classification of Diseases (ICD) diagnostic code Z59.0 in inpatient and outpatient records.	Descriptive diagnostic codes indicating “homelessness.”
CDW	Outpatient stop codes 504, 508, 511, 528, 529, 555, and 556. Inpatient specialty codes 28, 29, 37, and 39.	Clinical encounters with outpatient staff in the Grant and Per Diem (GPD) program, the Health Care for Homeless Veterans (HCHV)/Homeless Chronically Mentally Ill (HCMI) program, and the Homeless Veterans Community Employment Services (HVCES) program. Clinical encounters with inpatient staff included the Domiciliary Care for Homeless Veterans (DCHV) program and the Compensated Work Therapy/Transitional Residence (CWT/TR) program.
Homeless Operations, Management and Evaluation System (HOMES)	Record of participation in GPD, HCHV/HCMI, DCHV, or CWT/TR.	The GPD provides transitional housing in the community and the DCHV provides transitional housing at VHA facilities. The HCHV/HCMI program provides short-term case management and temporary housing. The CWT/TR program provides vocational rehabilitation and transitional housing.
CDW	A positive screen on part 2 of a two-part annual Homeless screening clinical reminder (HSCR).	A “no” response to the question: “In the past two months, have you been living in stable housing that you own, rent, or stay in as part of a household?”

## References

[1] Tsai J, Szymkowiak D, Jutkowitz E. Developing an operational definition of housing instability and homelessness in Veterans Health Administration’s medical records. PLoS One. 2022;17(12):e0279973. doi: 10.1371/journal.pone.0279973 36584201 PMC9803152

