# Supplementary material for: Developing an operational definition of housing instability and homelessness in Veterans Health Administration’s medical records
Source: PLoS One. 2022 Dec 30;17(12):e0279973. doi: 10.1371/journal.pone.0279973 (PMC9803152; doi:10.1371/journal.pone.0279973)
Supplement: S1 Fig — Note: ICD = International Classification of Diseases; HSCR = Homeless Screening Clinical Reminder; HUD-VASH = Housing and Urban Development-Veterans Affairs Supportive Housing, HOMES = Homeless Operations Management Evaluation System, SSVF = Supportive Services for Veterans Families, RRH = Rapid Re-housing. (DOCX) [file pone.0279973.s001.docx]

S1 Figure. Numbers of unstably housed veterans identified with different data sources, treating participation in HUD-VASH as an indicator of housing instability


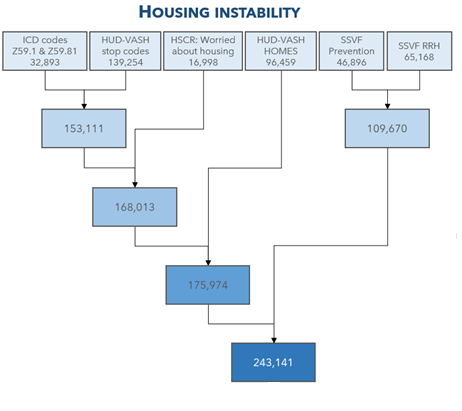


Note: ICD= International Classification of Diseases; HSCR= Homeless Screening Clinical Reminder; HUD-VASH= Housing and Urban Development-Veterans Affairs Supportive Housing, HOMES= Homeless Operations Management Evaluation System, SSVF= Supportive Services for Veterans Families, RRH= Rapid Re-housing.
