# Supplementary material for: Developing an operational definition of housing instability and homelessness in Veterans Health Administration’s medical records
Source: PLoS One. 2022 Dec 30;17(12):e0279973. doi: 10.1371/journal.pone.0279973 (PMC9803152; doi:10.1371/journal.pone.0279973)
Supplement: S1 Table — Note: A map of VISN locations can be viewed here: https://www.va.gov/HEALTH/visns.asp ICD-10 = International Classification of Diseases, Version 10; HSCR = Homeless Screening Clinical Reminder; HOMES = Homeless Operations Management Evaluation System; SSVF = Supportive Services for Veteran Families; HMIS = Homeless Management Information System. (DOCX) [file pone.0279973.s002.docx]

S1 Table. Geographic variability in use of different indicators of housing instability

| **Veterans Integrated Services Network (VISN)** | **ICD-10 diagnostic codes**  **% of total homeless veterans in VISN** | **HSCR**  **% of total homeless veterans in VISN** | **Used SSVF prevention services as recorded in HMIS**  **% of total homeless veterans in VISN** | **Used SSVF rapid rehousing services as recorded in HMIS**  **% of total homeless veterans in VISN** | **Used any SSVF services as recorded in HMIS**  **% of total homeless veterans in VISN** | **Total number of unique homeless veterans identified across indicators** |
| --- | --- | --- | --- | --- | --- | --- |
| 1 | 22.3% | 9.9% | 31.4% | 45.2% | 75.4% | 5,997 |
| 2 | 22.8% | 9.0% | 39.4% | 35.3% | 73.6% | 7,536 |
| 4 | 22.3% | 9.8% | 39.1% | 35.8% | 73.5% | 6,528 |
| 5 | 19.7% | 9.9% | 35.5% | 43.6% | 77.0% | 6,342 |
| 6 | 22.1% | 15.8% | 25.8% | 42.3% | 67.5% | 6,512 |
| 7 | 33.0% | 13.5% | 30.6% | 31.0% | 61.0% | 9,268 |
| 8 | 21.0% | 9.3% | 37.7% | 40.0% | 75.9% | 13,436 |
| 9 | 20.8% | 9.4% | 37.7% | 38.9% | 74.6% | 5,085 |
| 10 | 23.7% | 10.4% | 33.4% | 40.5% | 72.1% | 10,491 |
| 12 | 28.2% | 10.8% | 28.7% | 40.0% | 67.0% | 5,222 |
| 15 | 36.8% | 10.6% | 28.7% | 33.2% | 61.2% | 4,668 |
| 16 | 16.9% | 10.7% | 36.9% | 41.7% | 77.1% | 9,164 |
| 17 | 24.6% | 17.4% | 32.4% | 32.7% | 64.1% | 8,552 |
| 19 | 16.0% | 10.7% | 34.5% | 45.5% | 78.4% | 9,671 |
| 20 | 15.4% | 9.8% | 24.1% | 55.4% | 78.8% | 8,812 |
| 21 | 21.3% | 9.9% | 22.7% | 54.7% | 76.4% | 13,191 |
| 22 | 20.1% | 12.3% | 17.3% | 56.8% | 73.2% | 16,914 |
| 23 | 11.2% | 12.6% | 40.5% | 40.4% | 79.4% | 4,598 |
| Total (across VISNs) | 21.9% | 11.3% | 31.3% | 43.4% | 73.1% | 149,991 |

Note: A map of VISN locations can be viewed here: <https://www.va.gov/HEALTH/visns.asp> ICD-10= International Classification of Diseases, Version 10; HSCR= Homeless Screening Clinical Reminder; HOMES= Homeless Operations Management Evaluation System; SSVF= Supportive Services for Veteran Families; HMIS= Homeless Management Information System.
